# An illustrative case of hypopigmented mycosis fungoides

**DOI:** 10.1177/2050313X251359024

**Published:** 2025-07-31

**Authors:** Karen Michael, Annie R. Langley

**Affiliations:** 1 University of Western Ontario Medical School, Windsor, ON, Canada; 2 Division of Dermatology, Department of Medicine, University of Ottawa, ON, Canada

**Keywords:** mycosis fungoides, cutaneous T cell lymphoma, hypopigmentation

## Abstract

Hypopigmented mycosis fungoides (MF) is a rare variant of mycosis fungoides that creates diagnostic challenges as it can mimic other more common hypopigmented skin conditions clinically. Herein we present a case of a 31-year-old female with hypopigmented MF with typical histopathological features and excellent response to narrow-band ultraviolet B phototherapy combined with mid-potency topical corticosteroids.

## Introduction

Primary cutaneous T cell lymphomas are a subtype of non-Hodgkin lymphoma. Mycosis fungoides (MF) is the most common type of cutaneous T cell lymphoma.^1^ It originates from memory T cells which express T cell receptor and CD4+ immunophenotype.^1^ The etiology of this condition is not well known but factors including environmental, genetic, and infectious agents have been reported.^2^

There are several variants of MF including classic Alibert-Bazin type, folliculotropic, pagetoid reticulosis, granulomatous slack skin, and hypopigmented.^2^ MF is often diagnosed late in disease course due to similarities in appearance to other inflammatory skin disorders and nonspecific pathological features. This may be even more so for less common variants of MF due to their atypical appearance.

## Case report

**Figure 1.**
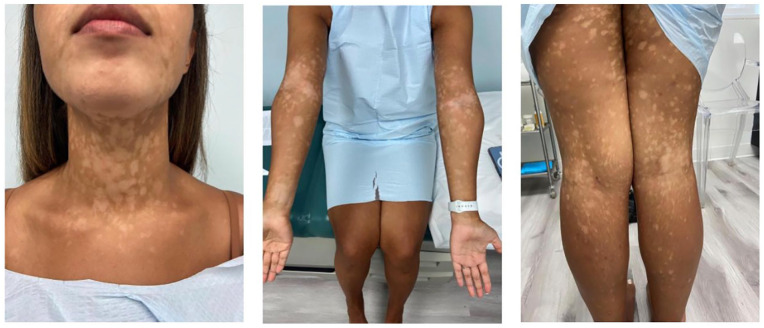
Well-demarcated hypomelanotic macules with subtle scale, coalescing into patches.

An otherwise healthy 31-year-old female presented to the dermatology clinic for a 1 year history of a hypopigmented rash. On exam, she was found to have well-demarcated hypomelanotic macules with subtle scale, coalescing into patches on her torso, neck, and arms (Figure 1). Woods lamp examination confirmed hypopigmentation. The favored diagnosis was tinea versicolour, other differential diagnosis included progressive macular hypomelanosis, postinflammatory hypopigmentation, vitligo, hypopigmented sarcoid, and hypopigmented mycosis fungoides. Initial skin scraping was positive for malasezzia, and she was treated for tinea veriscolor with standard topical and oral antifungal agents. A trial of treatment for progressive macular hypomelanosis with doxycycline 100 mg PO daily for 8 weeks similarly had no effect. A biopsy was performed showing epidermotropism of atypical small-to-medium sized hyperchromatic lymphocytes with irregular nuclear contours. There was dermal fibrosis and minimal spongiosis. Pautrier microabscesses were present. There was also mild to focally moderate lymphocytic inflammatory infiltrate within the papillar dermis. The intraepidermal CD2 and CD3 positive cells showed a CD4 to CD8 ratio of approximately 5 to 1. CD20 and CD30 were negative. The findings were suspicious for mycosis fungoides. Clinically, this correlated to a diagnosis of patch stage 1B hypopigmented MF given >10% body surface area involvement.

**Figure 2.**
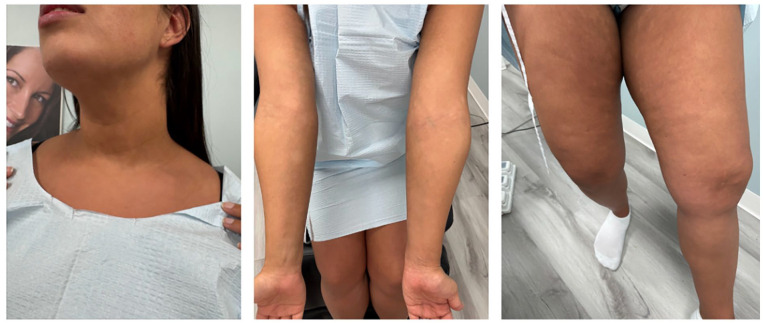
Near complete remission of hypopigmented macules after completion of 4 months of nb-UVB phototherapy.

The patient was treated with narrow band UVB (nb-UVB) phototherapy and mid-potency topical corticosteroids (betamethasone valerate 0.1% cream daily). She had significant improvement of her rash with near complete repigmentation of affected areas after 4 months of treatment (Figure 2).

## Discussion

Hypopigmented MF is a rare variant of cutaneous T-cell lymphoma characterized by mostly asymptomatic hypopigmented scaly macules and patches.^3^ Compared to classic MF which most commonly affects elderly white males, this variant typically occurs in younger female patients, often with skin of color.^3^ Similar to classic MF, distribution is predominantly over the trunk; however, this variant more commonly also affects the extremities and head.^3^ Features of hypopigmented MF on histopathology include little spongiosis, focal parakeratosis, and upper dermal lymphocytic infiltrate with coarse collagen bundles and intense epidermotropism. Pautrier microabscesses are less commonly noted.^3^ Epidermotropism is predominantly CD8+ T cells as opposed to CD4+ T cells more commonly seen in classic MF.^3^

Our case highlights the classic but unusual features of hypopigmented MF and the commonly affected demographic. Hypopigmented MF should be considered on the differential diagnosis for hypopigmented rashes such as tinea versicolor, and skin biopsy should be strongly considered if presentation is atypical or if treatment response is not as expected. Treatment options for hypopigmented MF include phototherapy (NB-UVB, PUVA), topical nitrogen mustard, topical corticosteroids, topical carmustine, and topical tazarotene.^4^ Commonly, phototherapy will be combined with topical corticosteroids as used here. Similar resolution of hypopigmented MF with nbUVB phototherapy has been documented previously.^5–7^
